# Supplementary material for: Invasive Stages within Alien Species and Hutchinson’s Duality: An Example Using Invasive Plants of the Family Fabaceae in Central Chile
Source: Plants (Basel). 2022 Apr 13;11(8):1063. doi: 10.3390/plants11081063 (PMC9029910; doi:10.3390/plants11081063)
Supplement: Supplementary file 1 [file plants-11-01063-s001.zip › plants-1621728-supplementary/history.pdf]

INVASION HISTORY OF SIX FABACEAE SPECIES IN CHILE

Acacia dealbata Link (Fabaceae)

This tree species, is native to the southeast coast of Australia, but which has colonized vast areas of South America [1]. In Chile, this species was introduced for ornamental purposes in 1869 [2]. Its distribution is concentrated mainly in the central-southern zone of the country, considered as a weed in forest plantations and also very abundant in pre-Andean basins of central Chile [3].

Teline monpessulana

This species is highly invasive species in the Mediterranean and temperate climates of the world [4]. Its origin is the Mediterranean basin. Currently it has colonized other regions of the world with climates very different to those of its native range [5]. In Chile, the first record of this species was recorded Chile in 1847 for ornamental uses [6]. Currently, its distribution range is from 32° to 40° S.

Cytisus striatus Rothm. (Fabaceae).

This a shrub, original from Iberian Peninsula; it grows in rocky and acidic soils at altitudes between 30 and 1400 meters [7]. In Chile, C. striatus was introduced in 1897 for ornamental uses [2]. Its current distribution is restricted from the Maule Region to the Araucanía Region [2].

Acacia melanoxylon R. Br (Fabaceae)

This species is a forest tree native from Australia [8]. It is original from the southeast of Australia and northwest of Tasmania island. IT has colonized a wide variety of climatic zones [9]. In Chile, this species was introduced in 1923, for ornamental use and furniture [2].

Lotus corniculatus L. (Fabaceae)

Es una especie arbustiva nativa de Eurasia occidental y el norte de África y que ha sido introducida alrededor del mundo como planta de forraje de alto nivel nutricional: Es reconocida como invasora en Estados Unidos y el Norte de Japón [10]. En Chile, su primer registro data de 1847 [2]. Su distribución en Chile va desde la Región Metropolitana a la Región de los Lagos.

Ulex europaeus L. (Fabaceae)

U. europaeus es un arbusto perenne nativo de la costa Atlántica de Europa y las Islas Británicas [11]. Fue introducido el año 1840 para ser usada como cerco vivo principalmente. Este arbusto está considerado por la IUCN como una de las 100 peores especies invasoras del mundo. En Chile, esta especie se distribuye entre los 37° y los 43° y afecta severamente a la agricultura y la silvicultura entre los 37° y los 43° S [12].

References:

1. Pauchard, A.; Maheu-Giroux, M. Acacia Dealbata Invasion across Multiple Scales: Conspicuous Flowering Species Can Help Us Study Invasion Pattern and Processes.; 2007; p. 203.

2. Fuentes, N. *Plantas Invasoras Del Centro-Sur de Chile: Una Guía de Campo*; Laboratorio de Invasiones Biológicas, 2014; ISBN 956-358-031-1.
3. Becerra, P.I. Invasión de Árboles Alóctonos En Una Cuenca Pre-Andina de Chile Central. *Gayana. Botánica* **2006**, *63*, 161–174.
4. García, R.A.; Pauchard, A.; Peña, E. Banco de Semillas, Regeneración y Crecimiento de Teline Monspessulana (L.) K. Koch Después de Un Incendio Forestal. *Gayana. Botánica* **2007**, *64*, 201–210.
5. García, R.A.; Pauchard, A.; Cavieres, L.A.; Peña, E.; Rodríguez, M.F. El Fuego Favorece La Invasión de Teline Monspessulana (Fabaceae) al Aumentar Su Germinación. *Rev. chil. hist. nat.* **2010**, *83*, doi:10.4067/S0716-078X2010000300011.
6. Matthei, O. *Manual de Las Malezas Que Crecen En Chile*; Editorial Universidad de Concepción: Concepción, Chile, 1995;
7. Rodríguez-Echeverría, S.; Pérez-Fernández, M. The Seasonal Impact of Cytisus Striatus on Soil Fertility and the Herbaceous Understory. *Journal of Mediterranean Ecology* **2003**, *4*, 35–43.
8. Hussain, M.I.; Gonzalez, L.; Reigosa, M.J. Allelopathic Potential of Acacia Melanoxylon on the Germination and Root Growth of Native Species. *Weed Biology and Management* **2011**, *11*, 18–28.
9. Ramírez, J.C.; Schlatter, J. Análisis de Variables de Sitio Para Estimar El Establecimiento En Chile de Acacia Melanoxylon R. Br. *Bosque* **1998**, *19*, 37–51.
10. Mimura, M.; Ono, K.; Goka, K.; Hara, T. Standing Variation Boosted by Multiple Sources of Introduction Contributes to the Success of the Introduced Species, Lotus Corniculatus. *Biological invasions* **2013**, *15*, 2743–2754.
11. Hill, R.; Ireson, J.; Sheppard, A.; Gourlay, A.; Norambuena, H.; Markin, G.; Kwong, R.; Coombs, E. A Global View of the Future for Biological Control of Gorse, Ulex Europaeus L.; Citeseer, 2008; pp. 680–686.
12. Altamirano, A.; Cely, J.P.; Etter, A.; Miranda, A.; Fuentes-Ramirez, A.; Acevedo, P.; Salas, C.; Vargas, R. The Invasive Species Ulex Europaeus (Fabaceae) Shows High Dynamism in a Fragmented Landscape of South-Central Chile. *Environmental monitoring and assessment* **2016**, *188*, 1–15.
