# Supplementary material for: Invasive Stages within Alien Species and Hutchinson’s Duality: An Example Using Invasive Plants of the Family Fabaceae in Central Chile
Source: Plants (Basel). 2022 Apr 13;11(8):1063. doi: 10.3390/plants11081063 (PMC9029910; doi:10.3390/plants11081063)
Supplement: Supplementary file 1 [file plants-11-01063-s001.zip › plants-1621728-supplementary/Supplementary material.pdf]

SUPPLEMENTARY MATERIAL

Table S3: Average AUC and Boyce indexes for Global and Regional SDMs and train/test data for a set of six invasive Leguminous species.

Species	Boyce index Train global model	Boyce index Train regional model	Boyce index Test global model	Boyce index Test regionall model	AUC Train global model	AUC Train regional model	AUC Test global model	AUC Test regional model
Acacia dealbata Link	0.986 ± 0.007	0.975 ± 0.018	0.947 ± 0.03	0.861 ± 0.10	0.933 ± 0.0002	0.941 ± 0.002	0.932 ± 0.002	0.925 ± 0.006
Acacia melanoxylon R. Br.	0.957 ± 0.026	0.959 ± 0.024	0.888 ± 0.05	0.771 ± 0.13	0.909 ± 0.0001	0.956 ± 0.002	0.907 ± 0.002	0.942 ± 0.007
Cytisus striatus (Hill) Rothm.	0.978 ± 0.007	0.933 ± 0.036	0.933 ± 0.02	0.717 ± 0.15	0.985 ± 0.0003	0.961 ± 0.003	0.981 ± 0.003	0.954 ± 0.012
Lotus corniculatus L.	0.991 ± 0.003	0.953 ± 0.023	0.985 ± 0.005	0.626 ± 0.21	0.765 ± 0.0001	0.952 ± 0.005	0.763 ± 0.002	0.940 ± 0.032
Teline monspessulana (L.) K. Koch	0.973 ± 0.015	0.973 ± 0.015	0.867 ± 0.06	0.809 ± 0.10	0.99 ± 0.0004	0.963 ± 0.003	0.987 ± 0.002	0.947 ± 0.007
Ulex europaeus Brot.	0.958 ± 0.022	0.964 ± 0.011	0.901 ± 0.05	0.862 ± 0.10	0.921 ± 0.0001	0.967 ± 0.002	0.920 ± 0.002	0.962 ± 0.007

Table S4. Proportion of presences (omission rate) not predicted by Global SDM's for a set of six invasive Leguminous species in Chile.

Species	Omission rate (%) of the average Global SDMs
Acacia dealbata Link	10.81
Acacia melanoxylon R. Br.	25.68
Cytisus striatus (Hill) Rothm.	16.13
Lotus corniculatus L.	1.61
Teline monspessulana (L.) K. Koch	6.84
Ulex europaeus Brot.	6.00

Table S5. Contribution (%) of bioclimatic variables for global and regional niche models, in six invasive leguminous species, Central Chile.

	**Contribution (%)**											
	**Global model**						**Regional model**					
**Specie**	**Bio 1**	**Bio 5**	**Bio 6**	**Bio 7**	**Bio 11**	**Bio 12**	**Bio 1**	**Bio 5**	**Bio 6**	**Bio 7**	**Bio 11**	**Bio 12**
Acacia dealbata Link	10.5	14.9	21.1	21.1	26.5	6.0	44.5	4.5	2.8	1.4	9.5	37.3

Cytisus striatus (Hill) Rothm.	24.3	2.8	38.7	12.7	9.1	12.4	1.0	30.1	1.4	9.8	25.2	32.5
Teline monspessulana (L.) K. Koch	24.1	2.0	17.6	11.5	33.9	11.0	17.1	8.3	29.4	3.8	8.0	33.4
Acacia melanoxylon R. Br.	13.7	8.4	20.7	19.7	22.8	14.8	11.8	1.5	32.9	2.9	17.3	33.7
Ulex europaeus Brot.	30.0	0.2	28.8	32.9	1.5	6.5	1.1	0.7	2.6	3.4	55.9	36.3
Lotus corniculatus L.	36.1	1.8	1.8	1.0	29.9	29.4	0.2	20.9	1.2	10.4	28.8	38.6

Table S6. Potential area (km2) for Global and Regional SDMs for a set of six invasive Leguminous species.

species	Global model (km2)	local model (km2)
Acacia dealbata Link	132371	96651
Acacia melanoxylon R. Br.	110394	65600
Cytisus striatus (Hill) Rothm.	76521	63582
Lotus corniculatus L.	336963	106378
Teline monspessulana (L.) K. Koch	114371	63535
Ulex europaeus Brot.	136235	53879

Table S7: Proportion of plant populations falling inside four invasive stages proposed by Galliem et al. (2012) for six alien leguminous species. QE: quasi-equilibrium, LA: Local adaptation, COL: Colonization and SINK: Sink.

SPECIES	EQ	LA	COL.	SINK	TOTAL OCCURRENCES
Acacia dealbata Link	0.47	0.24	0.24	0.06	259
Cytisus striatus (Hill) Rothm	0.63	0.00	0.15	0.23	62
Teline monspessulana (L.) K. Koch	0.56	0.04	0.30	0.09	117
Acacia melanoxylon R. Br.	0.36	0.30	0.21	0.13	148
Ulex europaeus Brot.	0.50	0.08	0.22	0.20	100
Lotus corniculatus L.	0.60	0.02	0.16	0.23	62
**AVERAGE (RANGE)**	0.52 (0.36 - 0.63)	0.11 (0 - 0.30)	0.21 (0.15 – 0.30)	0.16 (0.15 - 0.23)	761 (62 – 259)

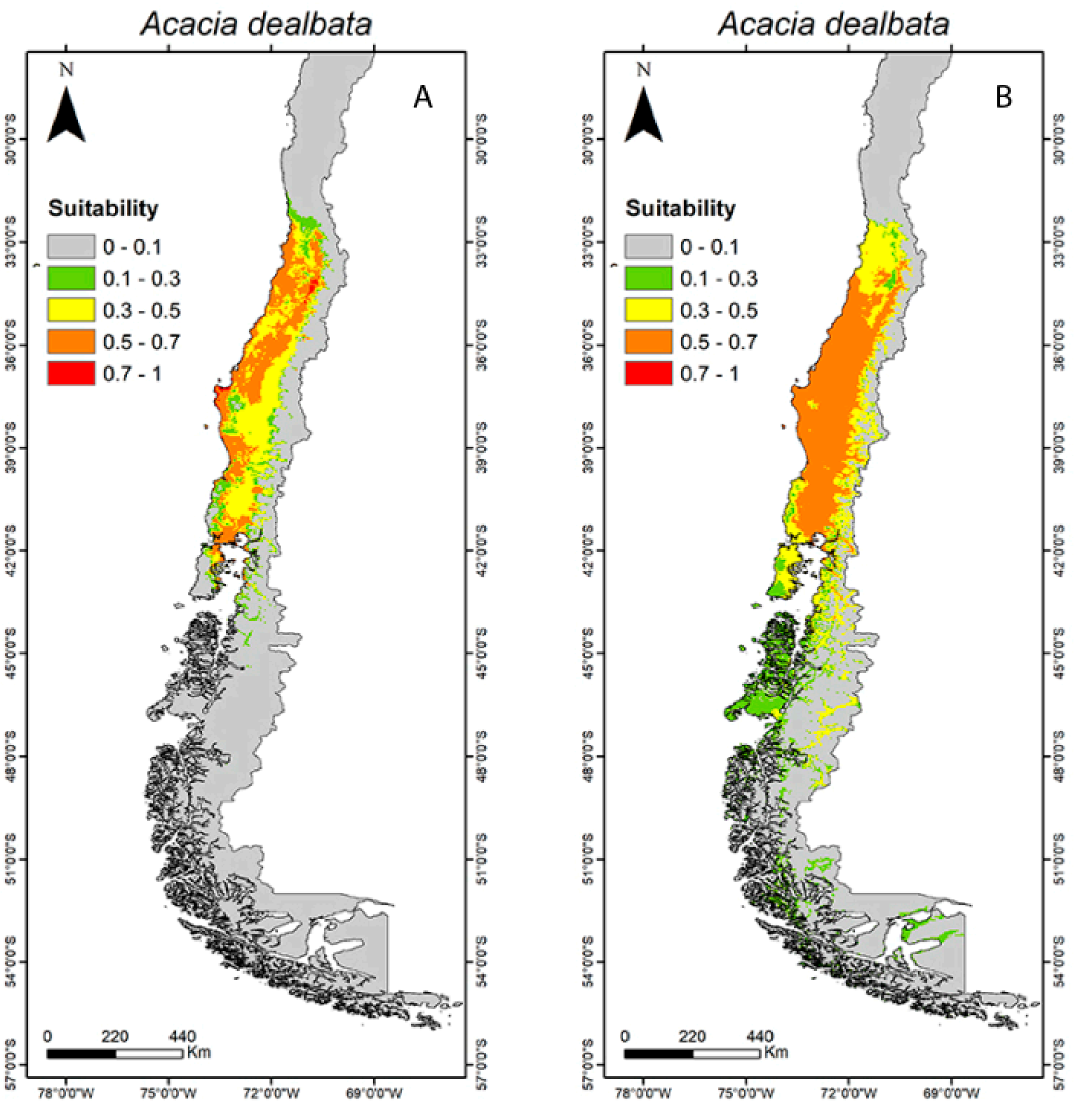

Figure S1.- SDMs predictions for *Acacia dealbata* Link in Chile: A) SDM projected from Regional niche model; B) SDMs projected from Global niche model.

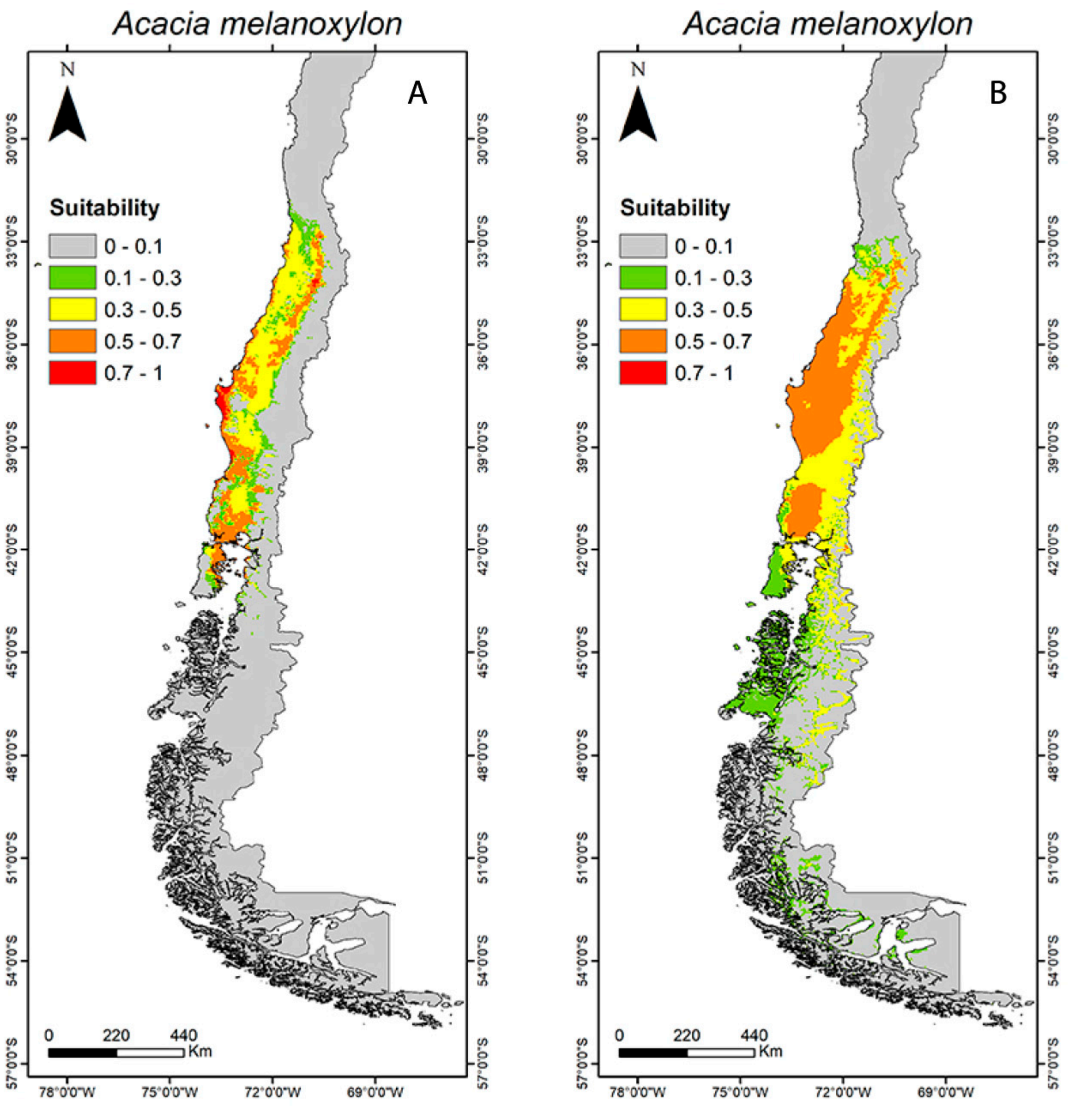

Figure S2.- SDMs predictions for *Acacia melanoxylon* R. Br. in Chile: A) SDM projected from Regional niche model; B) SDMs projected from Global niche model.

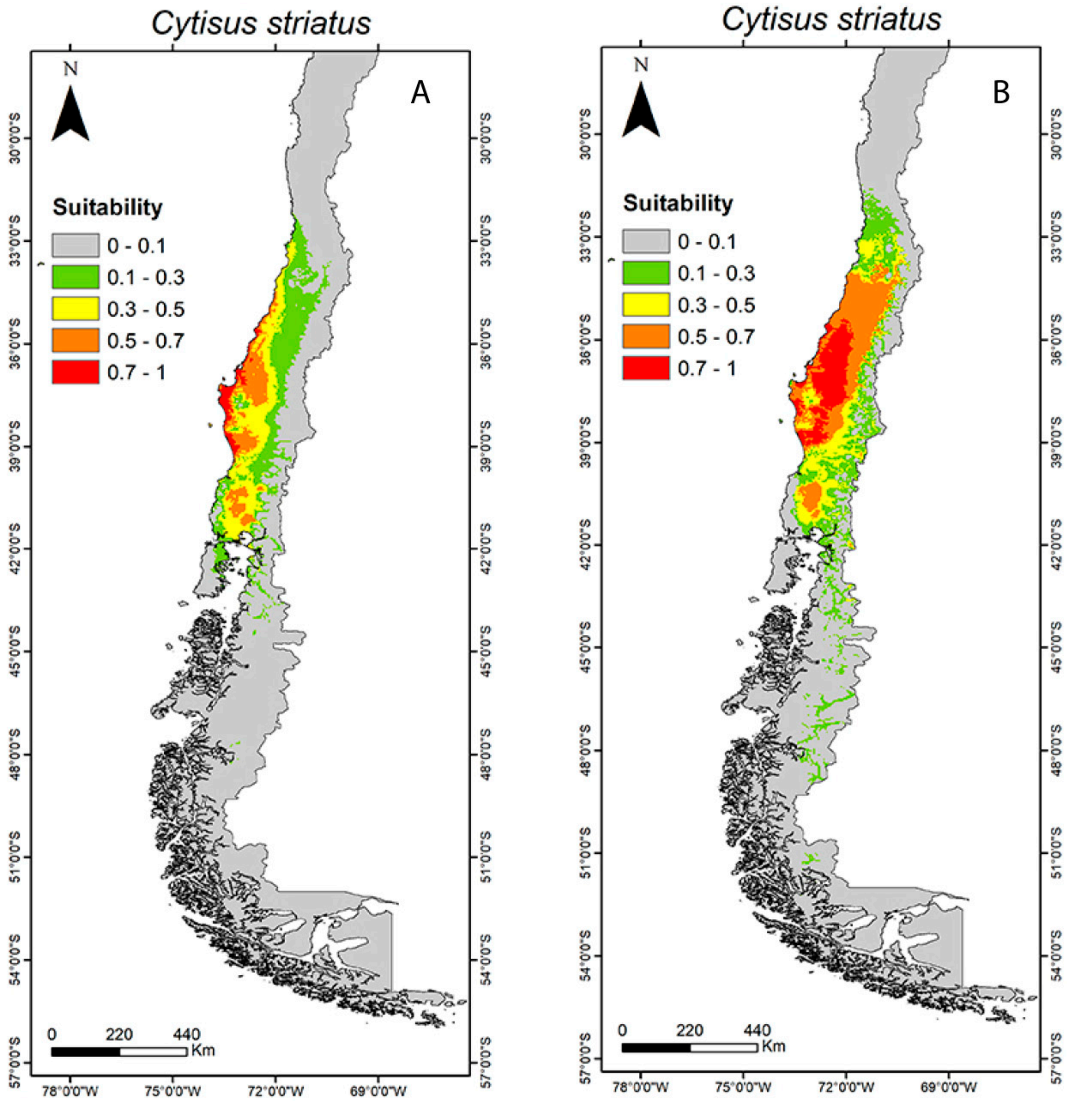

Figure S3.- SDMs predictions for *Cytisus striatus* (Hill) Rothm. in Chile: A) SDM projected from Regional niche model; B) SDMs projected from Global niche model.

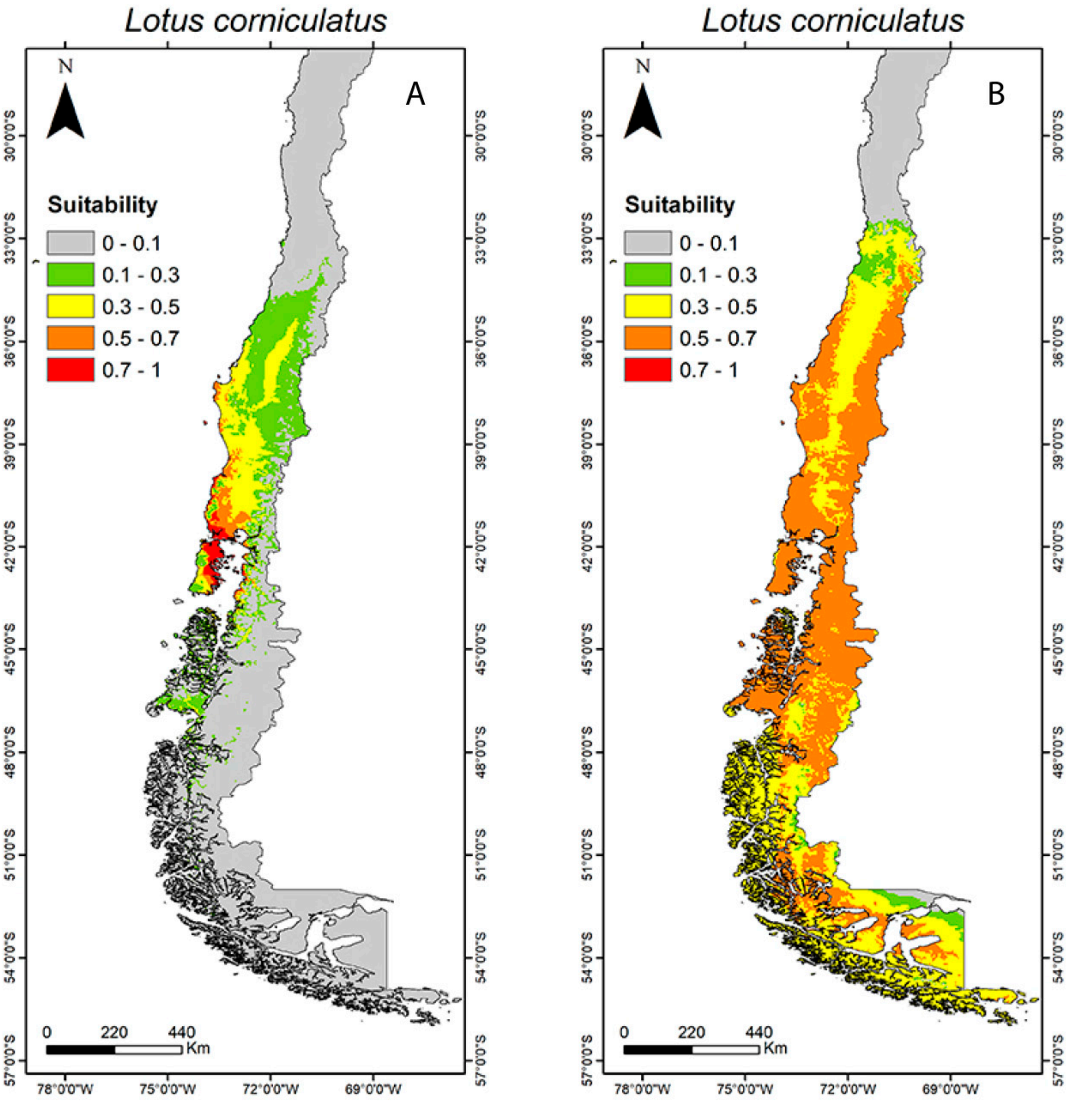

Figure S4.- SDMs predictions for *Lotus corniculatus* L. in Chile: A) SDM projected from Regional niche model; B) SDMs projected from Global niche model.

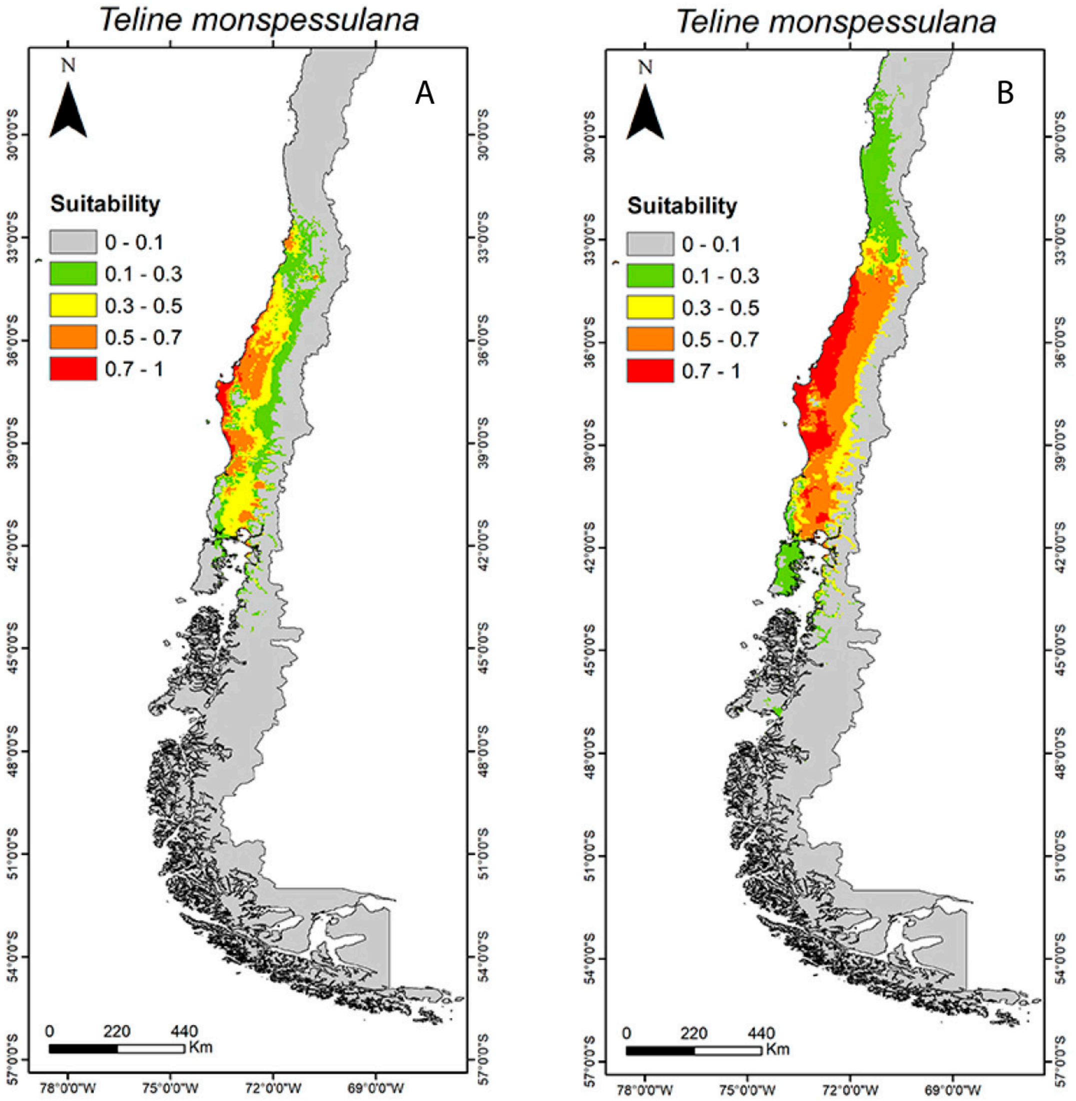

Figure S5.- SDMs predictions for *Teline monpessulana* (L.) K. Koch in Chile: A) SDM projected from Regional niche model; B) SDMs projected from Global niche model.

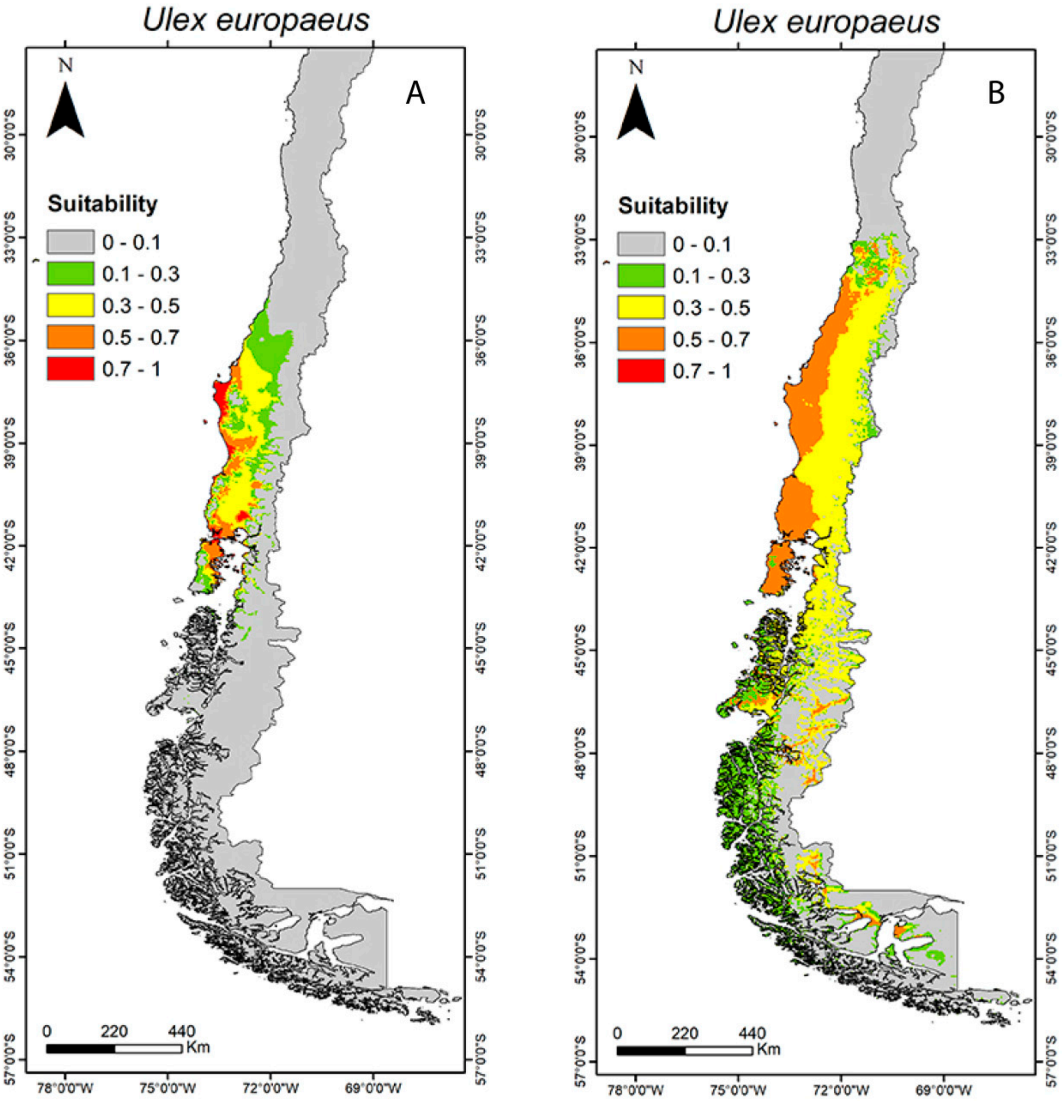

Figure S6.- SDMs predictions for *Ulex europaeus* Brot. in Chile: A) SDM projected from Regional niche model; B) SDMs projected from Global niche model.
